# Development of a Tool to Stage Households’ Readiness to Change Dietary Behaviours in Kerala, India

**DOI:** 10.1371/journal.pone.0165599

**Published:** 2016-11-18

**Authors:** Meena Daivadanam, T. K. Sundari Ravindran, K. R. Thankappan, P. S. Sarma, Rolf Wahlström

**Affiliations:** 1 Achutha Menon Centre for Health Science Studies, Sree Chitra Tirunal Institute for Medical Sciences and Technology, Thiruvananthapuram, 695011, India; 2 Dept. of Public Health Sciences (Global Health), Tomtebodavagen 18A, Karolinska Institutet, 171 77, Stockholm, Sweden; 3 Dept. of Food, Nutrition and Dietetics, Uppsala University, Box 560, SE-751 22, Uppsala, Sweden; 4 Family Medicine and Preventive Medicine, Dept. of Public Health and Caring Sciences, Uppsala University, Uppsala, Sweden; Northwestern University Feinberg School of Medicine, UNITED STATES

## Abstract

Dietary interventions and existing health behaviour theories are centred on individuals; therefore, none of the available tools are applicable to households for changing dietary behaviour. The objective of this pilot study was to develop a practical tool that could be administered by community volunteers to stage households in rural Kerala based on readiness to change dietary behaviour. Such a staging tool, comprising a questionnaire and its algorithm, focusing five dietary components (fruits, vegetables, salt, sugar and oil) and households (rather than individuals), was finalised through three consecutive pilot validation sessions, conducted over a four-month period. Each revised version was tested with a total of 80 households (n = 30, 35 and 15 respectively in the three sessions). The tool and its comparator, Motivational Interviewing (MI), assessed the stage-of-change for a household pertaining to their: 1) fruit and vegetable consumption behaviour; 2) salt, sugar and oil consumption behaviour; 3) overall readiness to change. The level of agreement between the two was tested using Kappa statistics to assess concurrent validity. A value of 0.7 or above was considered as good agreement. The final version was found to have good face and content validity, and also a high level of agreement with MI (87%; weighted kappa statistic: 0.85). Internal consistency testing was performed using Cronbach’s Alpha, with a value between 0.80 and 0.90 considered to be good. The instrument had good correlation between the items in each section (Cronbach’s Alpha: 0.84 (fruit and vegetables), 0.85 (salt, sugar and oil) and 0.83 (Overall)). Pre-contemplation was the most difficult stage to identify; for which efficacy and perceived cooperation at the household level were important. To the best of our knowledge, this is the first staging tool for households. This tool represents a new concept in community-based dietary interventions. The tool can be easily administered by lay community workers and can therefore be used in large population-based studies. A more robust validation process with a larger sample is needed before it can be widely used.

## Introduction

Dietary behaviour is the result of a complex interplay between food-related factors and other individual and environmental factors [1–3], which exert varying degrees of influence, depending on the context [4]. Similarly, dietary behaviour change involves more than choosing healthy foodstuffs; it includes making the decision to change and the actual process of change itself [1, 2, 5].

Health behaviour theories like the trans-theoretical model (TTM) and the social cognitive theory have been used as theoretical frameworks to understand food choice issues; predict dietary behaviour; develop interventions to change food habits; and facilitate the behaviour change process [5–7]. The TTM is the most widely used change model that describes the process of change, its initiation and maintenance [2, 8, 9]. It has been most commonly used to stage individuals for predicting potential for behaviour change or delivering stage-matched interventions [7]. The evidence regarding effectiveness of stage-matched interventions is mixed [9, 10]. However, it provides a method of identifying individuals at similar levels of willingness and motivation to change, which allows for more focussed intervention approaches.

Health behaviour theories, including TTM, are centred on the individual making the choice [11]. Consequently, existing staging tools or algorithms based on TTM are also focussed on individuals [5], and cannot be directly applied to households (HHs) as a whole. It therefore becomes a challenge regarding food choices and behaviour change, as decision-making is partly influenced by all individuals in the family by virtue of various power relations, and also by society [12]. In the context of rural Kerala in India, we have already found that dietary decisions are taken at the household level and that costs were the primary consideration, money costs in particular [13, 14]. The hierarchy of household members in terms of food preferences and the value ascribed to various foodstuffs were more important than their perceived health value [13]. Irrespective of their employment status, women’s identities, also strongly embedded as housewives and mothers had the primary responsibility to keep “husbands and children well fed” [15] (p. 266). Hence, the responsibility for cooking may rest with the women, but the decisions are often dictated by the preferences of spouse and children [13, 14].

The collective nature of the process of changing dietary behaviours may also make it difficult to predict the degree of success of individual-based staging tools or algorithms to assess the stage-of-change at HH level. This calls for modifying existing theoretical models to account for this shift from individual to collective decision-making [13, 14]; and development of tools that can be applied to HHs rather than individuals.

Two major literature reviews have been published looking specifically at TTM and dietary behaviour [5, 7]. Spencer et al identified 65 original studies published between 1999 and 2006. There were more than 35 staging algorithms or tools from these 65 studies to assess an individual’s stage of change [7]. Two of the most commonly used or modified staging algorithms have been the ones developed by Greene and Rossi [16] and Laforge et al [17] for low-fat diet and fruit and vegetable (FV) intake, respectively. Staging algorithms also seem to be “culturally and demographically specific” [7] However, to the best of our knowledge, there are no tools taking into account collective decision-making that would enable assessment of the stage-of-change for a family or household or a group.

Therefore, this was a pilot study that aimed to develop and validate a tool to stage households based on their readiness to change dietary behaviour, which could be administered by lay volunteers in community-based interventions.

This study was part of the formative research for a community-based dietary behaviour change intervention for prevention of chronic non-communicable diseases in rural Kerala, a pragmatic randomized controlled trial [18]. The tool was used to stage each household’s willingness to change behaviour, in order to deliver matched intervention strategies. The stage-of-change was re-assessed six months into the intervention period and strategies were changed to match the new stage. The intervention focused five dietary components: fruits, vegetables, salt, sugar and oil; based on convincing evidence linking diet to the risk of non-communicable diseases [19–21], and prevalent dietary practices of the study population [13, 18].

## Methods

### Study setting

Kerala follows a de-centralised system of administration at the state, district, block- and *grama- panchayat* or municipality (rural or urban administrative units) levels; with the latter being further divided into smaller administrative units called wards [22]. Thiruvananthapuram district with a population of about 3.3 million inhabitants was the overall setting of the study; conducted and monitored by the Achutha Menon Centre for Health Science Studies, a public health institution situated within the district. We selected the study population with the objective of ensuring that they were representative of the participants in the pragmatic randomized controlled trial [18], but not actively participating in the same. The present study was conducted in two adjoining grama panchayats not selected for the trial, with the first one selected randomly. Kerala has a well-developed and functioning women’s self help group network called the *Kudumbasree*, which is organised in the form of neighbourhood groups or *ayalkootams*. Three *ayalkootams* with predominantly middle-income HHs were identified randomly within the selected grama panchayats from *Kudumbasree* registers and approached through local leaders or elected representatives of the respective wards with invitations to participate in the study. The findings from two qualitative studies conducted prior to this study [13, 14], showed that money was a primary concern in the food decision-making process. They also showed that households belonging to the lowest socio-economic strata had very limited room for manoeuvring their household finances to meet even basis needs. Hence, it was felt that subsidies or special arrangements would have to be made if we were to include this group in the subsequent intervention trial [18]. As that was not feasible, this group was not included in the present study as well as the subsequent cluster randomized controlled trial [18].

### Study participants

The participants (N = 80) were female heads of the households and any other member of the household who they considered as being a major decision-maker related to food matters. The additional members who came along with a few of the participants included: husband (one case), mother (two cases), daughter (one case), mother- or daughter-in-law (two cases each). Previous work done by the authors had shown that usually the female head of the HH took dietary decisions, based on the stated and unstated preferences of other HH members, particularly spouse and children. In the previous study study, qualitative data from in-depth interviews and focus group discussions were analysed using manifest and latent content analysis to answer the question: How are food decisions made in households in rural Kerala? [13] The female head was the person who was most aware about the habits and preferences of all the HH members and therefore, a better judge of HH’s dietary consumption practices as well as their possible readiness to change.

### Data collection

#### The staging tool

**Fig 1:**
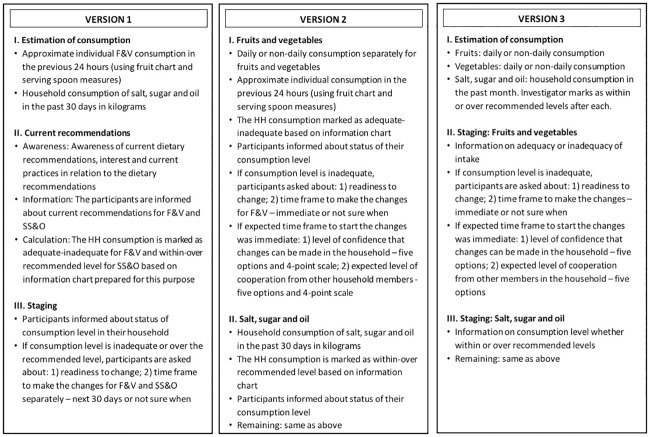
Differences between the three versions of the Household Staging tool. Fig 1 describes the differences between three versions of the Household Staging tool with details of their structure and components.

**Fig 2:**
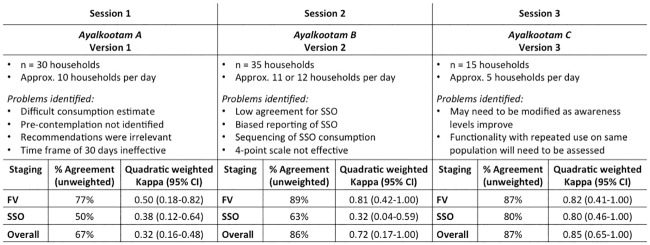
Results of the validation process for the Household Staging tool. Fig 2 shows the results of the validation process in terms of the Kappa score and percent agreement between the three versions of the Household Staging tool and Motivational Interviewing. Also, briefly outlines the problems identified while testing each version of the Household Staging tool. Abbreviations: FV: fruits and vegetables; SSO: salt, sugar and oil; CI: confidence interval.

The staging tool essentially consisted of a questionnaire that could be administered by a community volunteer, and an algorithm that could be used by the study team to compute the readiness to change based on the responses. Information regarding FV intake and salt, sugar and oil (SSO) consumption were collected through separate questions and then combined using the algorithm to compute the stage-of-change separately for FV and SSO. However, for stage matching and intervention delivery, it was essential that we had one overall stage for the HH. So, the FV and SSO stages were combined to form the overall stage. The actual process was much more complex, sometimes testing two or three algorithms at the same time. To avoid confusion, we have described only the best algorithm for each version in this paper (Table 1, S1 File). When the questionnaire was administered, the participant responded on behalf of the household, based on their daily practice. The stage-of-change was therefore computed for the household and not the specific participant. Three versions (Versions 1, 2 and 3) were developed consecutively, the third being the final one; with each subsequent version incorporating changes based on lessons learnt from the previous version (Figs 1 and 2). For the purpose of this study, we modified the original five stages of the TTM [23] to three stages as follows: 1) pre-contemplation, which was the same as the original pre-contemplation; 2) intention, which combined contemplation and preparation stages; and 3) action, which combined action and maintenance stages. This was based on our initial formative research, where we developed the conceptual model for the behaviour change intervention, which was tested in the pragmatic randomized controlled trial [14, 18]. Similar modifications have been used in other studies [24, 25]. The themes used to develop the components of the instrument and the algorithm (Table 1), and the decision to use three instead of five stages-of-change were derived from existing staging tools [24, 26–28], other studies who have used modified stages-of-change [23, 24] and the findings of a modified framework analysis of our own primary qualitative data [14].

**Table 1:** Algorithm components and identification of stage-of-change by version of the household staging tool.

Components incorporated in the staging algorithm to assess household stage-of-change	Relevant theme addressed by the component ^a^	Relevant stage identified in each version of staging tool ^c^		
		Version 1	Version 2	Version 3
**I. Estimation of consumption**				
• Daily intake of FV (Yes, No)	Self-evaluation	-	Yes = A	Yes = A
• HH consumption of SSO: within limits (WL) or outside ^b^	Self-evaluation	-	WL = A	WL = A
**II. Current recommendations**				
• Interest in current dietary recommendations (Yes, No)	Awareness	No = P, Yes = I	-	-
• Already following recommendations: FV (Yes, No)	Awareness	A	-	-
• Already following recommendations: SSO (Yes, No)	Awareness	A	-	-
**III. Staging questions**				
• Willingness to make changes (Yes, No)	Perceived HH response	No = P, Yes = A	No = P	No = P
• Time frame for change: unsure	Time frame for change	I	P	P
• Time frame for change: 30 days	Time frame for change	A	-	-
• Time frame for change: immediate	Time frame for change	-	I	I
• Level of confidence to make changes (5 options)	Household efficacy	-	1–4 = P, 5 = I	1–4 = P, 5 = I
• Level of expected cooperation (5 options)	Perceived HH response	-	1–3 = P, 4–5 = I	1–3 = P, 4–5 = I
**IV. Overall stage of the household**				
• Pre-contemplation (P)		FV & SSO = P	FV or SSO = P	FV or SSO = P
• Intention (I)		FV or SSO = I	FV or SSO = I	FV or SSO = I
• Action (A)		FV or SSO = A	FV & SSO = A	FV & SSO = A

Abbreviations: HH: household; FV: fruits and vegetables; SSO: salt, sugar and oil.

^a^ All themes were derived from the results of a modified framework analysis carried out on primary qualitative data (Daivadanam et al. BMC Public Health 2014;14:574), except ‘time frame for change’, which was based on existing staging tools.

^b^ Assessed using a tabular chart showing the recommended levels of consumption for SSO by HH size, modified for the local context from the dietary guidelines provided by the National Institute of Nutrition, Hyderabad, India.

^c^ The household stage corresponding to components in sections I, II and III relate to both FV and SSO, while section IV relates to the overall stage-of-change for the household. Only the responses definitively leading to a staging decision are indicated in this table.

#### Comparator

Motivational interviewing (MI), which is essentially a counselling approach [29, 30], was used as the comparator for assessing concurrent validity. We developed a format for a standardized procedure to administer MI (S2 File), with the aim of minimising subjective errors. This format was prepared in English to facilitate the process and it was verified and modified by one of the co-authors (RW), before translation to the local language, Malayalam. It was based on the Brief MI format [31] and inspired by a similar tool developed by Berg-Smith et al for a dietary intervention study in adolescents [24]. The format was meant to facilitate and standardize the encounter as much as possible, while sequence and content was modified to suit each participant. The interviews were conducted by the first author (MD), who had been trained in MI.

#### Tool accompaniments

Versions 1 and 2 of the staging tool were accompanied by serving spoons that measured one serving of cooked vegetables; and a fruit chart depicting one-serving size of the fruits commonly available in the local context. These were on display, and the participants were asked to estimate the intake of FV by an average adult in their household in the previous 24 hours. As the subsequent intervention study focused on adults in the household, the tool also elicited responses regarding FV intake by adults in the household. FV intake was assessed as adequate or inadequate by the interviewer based on the daily recommendation of five servings (modified to two fruit and three vegetable servings) [32, 33]. The participants were also asked to estimate the consumption of SSO in their household in the past 30 days. As FV was usually purchased on a daily, biweekly or weekly basis in the household, FV consumption was collected for the previous 24 hours to minimise recall bias. SSO on the other hand, was usually procured on a monthly or bimonthly basis and was used during food preparation and not at the point of consumption. Hence, respondents were clearly able to state how much was purchased and consumed in the last month. All three versions had a tabular chart for the interviewer to assess the household’s consumption of SSO as within-limits or over-the-limits. The tabular chart showed the recommended levels of consumption for SSO by HH size, modified for the local context from the dietary guidelines provided by the National Institute of Nutrition [34].

#### The process

The development and pilot validation of the household staging tool were conducted more or less simultaneously over a four-month period (February-May 2011). The first version of the household staging tool was developed in English and translated to the local language, Malayalam by the first author and checked for accuracy of meaning by a native speaker. All subsequent modifications were carried out on the Malayalam version itself. The validation process was carried out over three consecutive sessions (Fig 2) to compare the stage-of-change identified by the tool against the stage-of-change identified by MI. All members of the selected *ayalkootams* were invited to participate in the process on pre-arranged days and all those who came were included. Members from one *ayalkootam* were tested in one session (Fig 2). First, a research assistant administered the staging tool in the local language, followed by MI conducted by the first author (MD), in separate rooms. The research assistant estimated the consumption of FV and SSO as part of the staging tool; and only this was conveyed to the interviewer before the MI to avoid repetition. The interviewer independently assessed the stage-of-change for each of the households. Moreover, the stage computation using the algorithm was done only after completion of the testing of each version to prevent bias in stage assessment with MI. The administration of the tool took about 20 minutes for Versions 1 and 2 and about 10 minutes for Version 3, while the MI took about 30–40 minutes. Therefore, only 10–12 participants could be assessed on one day.

The time between two validation sessions was used to analyse the results and modify the questionnaire and the algorithm before the next testing. During each session, a new improved version of the staging tool was tested against MI using the same process as described above. Since the time period between each testing session was short (about 2–4 weeks only), we did not use the same population, as the likelihood was assumed to be high, that they would respond based on their memory of the previous session rather than the actual practice in their household.

It had not been the initial intention of the authors to carry out a three-stage validation process (Fig 2). However, after testing Version 1, it was clear that it was not functional, and therefore needed serious re-working. For this purpose, we revisited our primary qualitative data, where we identified HH efficacy and HH cooperation as two key components in HH food decision-making [14]. They were added to Version 2 and tested in the second session. Subsequently that version was also re-arranged based on the results of session 2; tested in session 3; and finalized.

### Assessing validity

#### Face and content validity

Colleagues assessed face and content validity of Version 3 of the tool. We conducted three rounds of iterations. The first was with the faculty members from the Achutha Menon Centre for Health Science Studies. They were invited to a formal meeting to discuss the first draft version of the tool using a checklist. The checklist covered the four components covered by the tool: 1) Estimation of consumption; 2) Current recommendations; 3) Staging questions; and 4) Overall stage of the household (Fig 1 and Table 1). The checklist and the tool were circulated before hand with instructions and inviting feedback. The tool was discussed at the formal meeting and notes were taken. In addition, written feedback was also collected. The modified version was then tested with two co-authors (RW and TKSR) in two subsequent rounds. During these rounds, TKSR specifically assessed the overall content and structure of the tool, and RW the questionnaire and its algorithm. Three field workers subsequently assessed Version 3, pilot-tested them among 7–10 households, and suggested modifications, based on the responses received.

#### Concurrent validity

The level of agreement between the stages identified by the staging tool and MI was tested using quadratic weighted Kappa statistic. As our stage variable is ordinal and the quadratic weighted Kappa is more sensitive in instances when a test fails to identify one or more categories, it was considered more appropriate for this study [35]. The analysis was carried out for a specified number of cases (Version 1 = 30; Version 2 = 35 and Version 3 = 15), three categories (stages-of-change: pre-contemplation, intention and action), and two tests (staging tool and MI) were specified. A value of 0.7 or above was considered as good agreement (Fig 2).

### Assessing reliability

Tests of reliability were restricted to assessing internal consistency using Cronbach’s alpha for the final version [36, 37]. Moreover, only one person computed the stages-of-change for all participants in the main study using the algorithm, hence tests for inter-rater reliability were also not carried out. A value of co-efficient of reliability, Cronbach’s Alpha between 0.8 and 0.9 was considered as indicating good agreement without a high level of item redundancy [38]. The final version had 17 distinct items: estimation of consumption (2-FV, 3-SSO items); staging questions (4-FV, 4-SSO items); the number of household members (1-common item); and stage computation (1-FV, 1-SSO and 1-overall item). For calculating Crohnbach’s alpha, the items were grouped into 8-FV items and 9-SSO items and 15-overall items (FV+SSO items, excluding 2-separate stage computation items). Cronbach’s alpha was then calculated separately for each of the three groups (FV, SSO and overall).

### Statistical analysis software

All statistical analysis was carried out using STATA data analysis software, version 12.1, owned by StataCorp, Texas, USA and licensed to Karolinska Institutet, Stockholm, Sweden.

### Ethics statement

This study was conducted according to the guidelines laid down by the Indian Council of Medical Research. The Institutional Ethics Committee of Sree Chitra Tirunal Institute for Medical Sciences and Technology, Thiruvananthapuram, Kerala, India approved all the procedures related to the study. All participants were invited for the validation-piloting process through the governing body of the respective *ayalkootams*. They were informed about the context, purpose and process of the study and gave preliminary approval to approach their members. Verbal informed consent was obtained from each of the participants, witnessed by their respective three-member governing body.

## Results

### The final instrument

Three versions of the staging tool were developed, the last (Version 3) being the final instrument. The three versions mainly differed in their structure, components and algorithm, which can be seen in Fig 1 and Table 1. A detailed description of the three versions is also provided separately (S1 File). The final instrument (Version 3) had two parts: a questionnaire with its accompanying tabular chart for household SSO consumption; and the algorithm. The questionnaire in Version 3 had three sections: 1) estimation of household consumption; 2) staging questions for FV; and 3) staging questions for SSO.

The tool utilised a three-step algorithm, which used the responses to these questions to compute an overall stage-of-change for the household. Steps 1 and 2 computed FV and SSO stage respectively, while step 3 computed the overall stage-of-change for the household. Pre-contemplation and intention stages were computed using time frame for change and household willingness, efficacy and cooperation; while action stage used self-evaluation (see ‘relevant theme addressed by the component’ in Table 1). Households were identified as being in pre-contemplation stage separately for FV and SSO (steps 1–2) if they chose any of the following three options: 1) they were not willing to make any changes; 2) were willing to make changes but were unsure as to when those changes could be made; 3) had low levels of confidence *or* low levels of expected cooperation from other household members. They were in intention stage if: 1) they were willing to make changes immediately *and* had high levels of confidence *and* expected cooperation. They were in action stage if: 1) they were consuming FV daily *or* were within limits for SSO consumption. The overall stage-of-change for the household (step 3) was identified as the lower of either FV or SSO stages. Therefore, the overall stage was action, only if it was action stage for both FV and SSO.

### Validation of the instrument

#### Face and content validity

The questionnaire and algorithm were assessed to have good face and content validity.

#### Concurrent validity

This was assessed using quadratic weighted kappa statistic to test the agreement between the stages identified by the staging tool and MI. The scores along with the identified problems for each of the three versions are shown in Fig 2. There were a total of 80 participants in the validation process in three consecutive sessions. Version 3 was able to identify all three stages for FV, SSO and overall for the HH, in good agreement (87%) with the staging assessed through MI (Kappa score = 0.85). Version 3 of the questionnaire was also tested for internal consistency for FV, SSO and overall. The coefficient of reliability, Cronbach’s alpha was 0.84 for FV, 0.85 for SSO, and 0.83 for overall, indicating good correlation between the items.

## Discussion

The major finding of this study was that it was possible to construct a contextualized and validated staging tool for households, that could be administered by lay volunteers.

The main feature of the tool is its ability to compute the stage-of-readiness to change behaviour for a household rather than an individual. The Indian society, including rural Kerala, is predominantly collectivist in nature [39], where people are “especially concerned with relationships” and “are interdependent within their in-groups; give priority to the goals of their in-groups; shape their behaviour primarily on the basis of in-group norms,…” [39] (p. 909). One such in-group is the household and the tools developed to stage individuals, if used for a household may elicit misleading responses leading to erroneous staging. Hence, it was necessary to develop a tool to assess stages-of-change at a household level.

Different staging tools have used different methods and components to identify the stage-of-change. Berg-Smith et al used a “ruler”; others have used scales or different combinations of algorithms [7, 24]. Two critical components in most staging tools were ‘Intention to act’ and ‘time frame for change’ in terms of 30 days or six months [16, 27]. However, we found that at the household level, it was not useful to differentiate stages on those grounds. ‘Intention to act’ elicited a positive response from all households, while ‘time frame for change’ detected a difference only when we used ‘immediate’ and ‘not sure’ as response options instead of ‘30 days’ and ‘six months’, respectively. As the participants were required to give a more definitive response at that point in time, inability to do so led to the identification of some of the pre-contemplators. Pre-contemplators were the most difficult group to identify using the staging tool and the most crucial in terms of the intervention. While the pre-contemplation stage is clearly defined in theory and unmistakably identifiable during MI, it was not easy to transfer our understanding of the setting and the culture to a tool that would ultimately be administered by lay volunteers.

Similarly, addition of two key components, namely, household efficacy and perceived household cooperation, identified in our own related study using modified framework analysis [14]; achieved a distinct differentiation between the stages-of-change at the household level. In our view, this was the most important addition to the tool. The existing staging tools or algorithms for individuals neither incorporate self-efficacy nor commitment [24, 26–28]. It is probable that these two components are not as important to delineate stages at an individual level, as at the household level.

### Methodological considerations

One of the major components of this study was the use of MI as the comparator for testing concurrent validity. MI is “a directive client-centred counselling approach for initiating behaviour change” [40] (p. 835) that was first described for problem drinkers [29]. MI and TTM integrate well together and have been extensively used in behaviour change interventions, including dietary behaviour [24]. MI provides a semi-structured, but non-threatening and non-judgmental way to understand an individual’s motivations and barriers, while at the same time supporting self-efficacy [30]. We used the brief MI format that is ideal for time-constrained settings [31]. Here, the interviewer (MD) was required to assess the appropriate stage-of-change for the household; based on her encounter with the participant and their conversation touching on these areas. As the interviewer was familiar with the culture and the way of thinking and talking prevalent in the setting, she was able to understand the nuances, and form her own judgment about the stage-of-change of the concerned household.

The validation process carried out as part of this pilot study has some limitations. First, the sample sizes used to test each of the three versions was small. The focus was on developing a functioning tool for the upcoming trial and time considerations prevented a more robust validation process at the time. More rigorous validation will be necessary before this tool can be widely used. Second, the MI was conducted immediately after administering the staging tool. While we had taken care to ensure minimal bias from the perspectives of both the interviewer and the participant, it is possible that we could not do so completely. Third, the participant selection was through random selection of three *ayalkootams*, not individual participants. The objective of the sample selection for validation of the tool was to make the results, and thereby the tool, applicable to the participants of the main community-based dietary intervention [18]. Fourth, only one of the participants had brought her husband as the co-decision maker. While men as breadwinners had a greater influence within their households [13, 14], they were reluctant to come forward for issues perceived to be in the women’s domain. Moreover, the validation sessions were conducted between 9 a.m. and 5 p.m. on most days, which was not a convenient time for men’s participation. Finally, though we used a standardized format and procedure to administer MI, the interviewer’s judgement cannot be eliminated as contributing to the staging. The standardized format and procedure can minimise subjective errors, although they cannot be completely eliminated, so a certain degree of misclassification of an individual’s stage of change cannot be excluded.

The staging was based on the responses of the female head of the household. This approach was based on findings from our preceding qualitative work, which identified the female head of the household as a good proxy to understand household response to dietary behaviour and change [13, 14]. However, we are aware that there may be differences between households, and the interpretation of our findings may not be applicable in each single case. The lack of measurement charts and serving tools in the final version could also be considered a limitation. Version 1 of the tool over-estimated consumption of FV and consequently the action stage. So, in the second version, we included both daily and non-daily consumption and serving measures (Fig 1). We found that the stage computed by the algorithm was the same when we used the daily or non-daily option alone or together with the serving measures. The reason for this was the very low daily consumption of fruits and vegetables and the low awareness regarding non-communicable diseases and their prevention strategies [41]. Hence, for the final version, we discarded the serving tools. The algorithm also addressed fruits and vegetables as one category, even though daily consumption data was collected separately. As in most cultures, fruits and vegetables are typically viewed differently. However, consumption of both fruits and vegetables was very low in the target population and therefore addressed together.

Development of a new tool can never be a smooth process and therefore is subject to limitations. We do not claim to have developed the perfect tool and acknowledge that more work needs to be done to improve the present one. However, this was the first attempt to develop such a tool for staging households, and it could reliably be used to differentiate households by one of three stages. The algorithm combined the FV and SSO staging in the final step to derive a single stage in order to make it practical for lay volunteers to deliver stage-matched interventions even in a large community-based study. However, the design of the tool is such that the data could be used to identify the stage-of-change for FV and SSO separately if needed.

The final version is simple enough to be administered by community workers after adequate training. However, its functionality with repeated use on the same population needs to be assessed. It is also important to remember that even though Kerala is a high literacy state, the rural population is still largely naive in terms of chronic disease risk factors and prevention [41]. The tool may need to be modified as the baseline awareness of the study population improves and food consumption patterns change. The tool is also specifically developed for this context and setting and should be modified and validated, if used in other settings. Potentially erroneous staging of households is another limitation of the tool. With an overall agreement of 87%, there is still a 13% misclassification by the staging tool, assuming that the staging by MI is devoid of similar errors. Further work should be done to reduce misclassification.

In spite of these limitations, this tool presents as a new concept and its development has been informed by formative research, combining constructs of existing theories and findings from primary qualitative research [13, 14].

### Next steps

This tool requires more work before it can be widely used in population-based studies. First, a more robust validation process with a larger sample is needed. Additional research is also needed to see how stage-of-change assessment for the household would be influenced if other decision-makers in the household (e.g. the earning member) were also interviewed; and to assess its ability to delineate stages with changing awareness levels and food consumption patterns. For the tool to be useful in intervention studies, repeatability in the same population at different time intervals and with varying intensity of the intervention also needs to be assessed.

### Conclusions

The staging tool for assessing households’ readiness-to-change represents a new concept in developing community-based dietary interventions. It incorporates the collective nature of dietary decision-making and behaviour prevalent in the study setting, by integrating components related to efficacy and commitment at the household level. This type of tool can be easily administered by community workers and can therefore be used in large population-based studies, after appropriate contextual modifications and validation.

## Supporting Information

S1 File Three versions of the Household staging tool. S1 File provides detailed descriptions of the three versions of the Household Staging tool in terms of its structure, components and algorithm.
(DOCX)

S2 File Standardized brief MI format. File S2 File is the format developed to standardize the delivery and content of the brief MI used as comparator in the pilot validation process.
(PDF)
