# Supplementary material for: Development of a Tool to Stage Households’ Readiness to Change Dietary Behaviours in Kerala, India
Source: PLoS One. 2016 Nov 18;11(11):e0165599. doi: 10.1371/journal.pone.0165599 (PMC5115657; doi:10.1371/journal.pone.0165599)
Supplement: S1 File — provides detailed descriptions of the three versions of the Household Staging tool in terms of its structure, components and algorithm. (DOCX) [file pone.0165599.s001.docx]

**Supplementary File S1**

**Three versions of the household staging tool**

**Version 1**

*Structure*

- Version 1 had three sections: estimation of household consumption; followed by a section on current dietary recommendations; and questions related to staging.
- Each of the three sections had separate questions related to FV and SSO.

*Components:*

- The major components of Version 1 that aided in staging were: use of fruit charts and household measures to estimate consumption of FV and SSO (self-evaluation); assessment of awareness of dietary recommendations, and informing the participants about the level of consumption in their households (awareness); staging questions based on readiness to make changes (intention to change); and time frame to make them in terms of next 30 days or unsure (time frame for change).
- The first version was modelled on existing staging tools for an individual; and therefore included a willingness component at the HH level as a proxy for the traditionally used ‘intention to change’^[[1]](#footnote-1)^. Time frame for change is also a part of most existing staging tools, but is usually asked in terms of 30 days or six months^[[2]](#footnote-2)^. When we initially tried these two options, all the responses clustered around 30 days, completely ignoring the six-months option. So, we used 30 days and ‘not sure’ in Version 1.
- Awareness about dietary recommendations was found to make no difference at all. On the one hand, none of the participants were aware of the dietary recommendations; while on the other hand; everyone universally indicated their interest to know more about it. So, it was unable to identify a difference and therefore discarded in subsequent versions.
- We estimated the FV consumption using serving spoon measures and fruit chart in Version 1. It was time consuming and we got erroneously high consumption as compared to 24-hour dietary recall. In the next version we added an item on daily and non-daily consumption of FV to close this gap.

*Algorithm*

- In Version 1, pre-contemplation stage was computed using responses related to awareness and household willingness; intention and action stages using awareness and time frame for change.
- Households were identified as being in pre-contemplation stage if they showed no interest in understanding or following current dietary recommendations; intention stage if they were interested in following current dietary recommendation and; action stage if they were already following recommendations or were willing to take action in the next 30 days. The overall stage-of-change for the household was then designated as the higher of either FV or SSO stage.
- Version 1 completely missed the pre-contemplators. First, even though no one knew the dietary recommendations, everyone expressed an interest to know more about it. Second, overall stage-of-change was pre-contemplation only if the stages-of-change for both FV and SSO were pre-contemplation. SSO stage tended to be higher than FV stage for most of the households. As the participants were the female heads of the households, they were essentially responsible for food procurement and preparation. Hence, they were more comfortable with the idea of changing SSO consumption, which they perceived to be in their hands, as opposed to FV consumption, which to some extent depended on the individual members of the household. So, even if they expressed unwillingness to make changes in FV consumption, and got identified as a pre-contemplator, their overall stage would be that of intention, owing to a higher SSO stage.

**Version 2**

*Structure*

- Version 2 separated the two sections corresponding to FV and SSO. Each section had subsections on estimation of consumption levels and questions related to staging.

*Components*

- A component to assess daily or non-daily consumption of FV was added while the one on assessment of awareness of dietary recommendations was deleted. The daily and non-daily consumption of FV elicited a far more realistic reflection of actual practices. Moreover, the responses received with serving spoons and fruit charts did not add substantially to the accuracy of the estimation of consumption. Hence, for Version 3, we only retained the item on daily and non-daily consumption for estimation of FV consumption.
- Version 2 introduced household efficacy (level of confidence to make the changes in the HH) and household cooperation (proxy for commitment at the household level), two aspects of collective decision based on the findings from the modified framework analysis^[[3]](#footnote-3)^. These were tested using a four-point scale and a five-option question. The four-point scale was discarded in the subsequent version, as it had been difficult for the respondents (not shown in table 1).
- The time frame for readiness to change was modified to immediate instead of next 30 days.
- The placement of the different components in the staging questionnaire was also important. This was particularly true for Version 2, where we got a very low level of agreement for SSO. Since the questions and the algorithm followed the same pattern as that for FV, we expected to see the same high level of agreement for SSO that we got for FV. However, we noticed under-reporting of consumption in the SSO section, as compared to the general trend that we had been seeing so far. The SSO segment immediately followed the staging questions for FV, so it was possible that participants were adjusting SSO to socially desirable levels, having already realised that their consumption of FV was inadequate. This was confirmed with Version 3, when the change in sequencing of questions led to a higher level of agreement.

*Algorithm*

- In Version 2, pre-contemplation and intention stages were computed using time frame for change and household willingness, efficacy and cooperation; while action stage used self-evaluation.
- Households were identified as being in pre-contemplation stage separately for FV and SSO if they were not willing to make any changes; were willing to make changes but were unsure as to when those changes could be made; had low levels of confidence or low levels of expected cooperation from other household members. They were in intention stage if they were willing to make changes immediately and had high levels of confidence and expected cooperation. They were in action stage only if they were consuming FV daily or were within limits for SSO consumption.
- The overall stage-of-change for the household was identified as the lower of either FV or SSO stages. Therefore, the overall stage was action, only if it was action stage for both FV and SSO.

**Version 3**

*Structure*

- The major difference between Version 2 and 3 was in the organization of its components.
- Version 3 had three sections, one on estimation of household consumption and the other two on staging related questions separately for FV and SSO.
- The estimation of household consumption was done together for both FV and SSO before the staging questions were administered.

*Components*

- The four-point scale to assess the level of confidence to make the changes and level of cooperation expected from other household members was not found to be effective and hence deleted. So, only the five-option questions were retained.
- The estimation of consumption for FV was made easier by asking for daily or non-daily consumption and so, the serving spoons and fruit chart were discarded.

*Algorithm*

- There was no change in the algorithm from Version 2.

1. Berg-Smith SM, et al 1999. Health Educ Res 14: 399-410; Hargreaves MK et al 1999. J Am Diet Assoc 99: 1392-1399; Littell & Girvin 2002. Behaviour Modification 26: 223-273; Kristal AR, et al 1999. J Am Diet Assoc 99: 679-684. [↑](#footnote-ref-1)
2. Greene & Rossi 1998. J Am Diet Assoc 98: 529-534. [↑](#footnote-ref-2)
3. Daivadanam et al 2014. BMC Public Health 2014;14:574. [↑](#footnote-ref-3)
