# Supplementary material for: Development of a Tool to Stage Households’ Readiness to Change Dietary Behaviours in Kerala, India
Source: PLoS One. 2016 Nov 18;11(11):e0165599. doi: 10.1371/journal.pone.0165599 (PMC5115657; doi:10.1371/journal.pone.0165599)
Supplement: S2 File — File S2 File is the format developed to standardize the delivery and content of the brief MI used as comparator in the pilot validation process. (PDF) [file pone.0165599.s002.pdf]

Staging of households: Brief Motivational Interviewing format

This is the format was prepared based on the brief motivational interviewing counseling methods and used by the principal investigator (MD) to assess stages-of-change during the validation of the HH staging tool.

Establish rapport

Hi, I am ______________________________. (Introduce self to all the members present and get introduced in return.)

Opening statement

We have about 30-40 minutes. We can talk about diet and discuss the possibility of making some modifications in your family diet. The aim is to help you make your household diet healthier by discussing how ready your household is to make any changes and how you can do it together as a family. We can help you by giving you the right information and discussing with you any small steps that you might want to take to get started in this direction.

Give feedback

Ask open-ended questions to assess current eating behaviour of household members. Refer to estimation of consumption done while administering the staging tool.

You have just been asked about the use of five dietary components in your household. Would you be interested in more information on where your household stands with respect to dietary recommendations?

If YES: This is where you stand (*give feedback and other interpretive information*).

What do you feel about all this information?

Now that you have been told about the current levels of use in your household, what do you feel? Where do you see your household fitting in with all this?

Assess readiness-to-change

Now that you know where you stand with respect to the recommended levels of use; where does that leave you now? How do you see your diet affecting your household? Would you be interested in more information, in order to decide what you can do next or are you able to see what you can do as a family with the available information itself?

What are your concerns about diet and dietary behaviour in your household?

If an interest is expressed towards making changes: Where do you think you need to make these changes? Why do you feel so?

If uninterested: Can you tell me why you feel so?

If uninterested: →close the encounter

If YES, continue:

Let's talk about fruit and vegetable intake first:

- What are your concerns about increasing fruit and vegetable intake?
- Where would you like to start making changes first?
- Which change would you consider as easy? Why?
- Which change would you consider as the most difficult? Why?
- How seriously are you considering making changes to fruit and vegetable intake in your household?
- When (in days, weeks or months) do you feel you would be able to start making these changes in your household? What are your reasons for choosing that particular time frame?
- Are you ready to start tackling any of them right away? Why do you feel so?

Now let's talk about salt, sugar and oil consumption in your household:

- Which of these would you like to tackle first? Why do you say so?
- What are your concerns about salt (sugar or oil) consumption in your household?
- Which change would you consider as easy? Why do you say so?
- Which change would you consider as the most difficult? Why do you say so?
- How seriously are you considering making changes to salt, sugar and oil consumption in your household?
- When (in days, weeks or months) would you be able to start making these changes in your household? What are your reasons for choosing that particular time frame?
- Are you ready to start tackling any of them right away? Why do you feel so?

Continue → Unsure or ready (as applicable)

<u>Close the Encounter</u>

<u>If NO: Stage 1 (Pre-contemplation)</u>

Summarize the session: So, now you know the recommended levels of use of five dietary components that we have been talking about and also the average levels of consumption of each in your household. You feel that currently you are not ready to start make changes in this direction. How can we help you to reconsider? Would more information help you?

Is there anything else that you would like to ask before we conclude this session? Thank you.

<u>If YES, but UNSURE about starting now: Stage 2 (Intention)</u>

Summarize the session: So, now you know the recommended levels of use of five dietary components that we have been talking about and also the average levels of consumption of each in your household. You feel that currently you are aware of the importance of making changes but you are unsure as to when you can start make changes in this direction. How can we help you to get there sooner?

Support self-efficacy: I congratulate you for arriving at a decision to seriously consider making changes to your household dietary practices. When you are ready, you can take small steps initially. Don't try to do everything at once. Keep discussing and talking amongst your household members and support each other.
Thank you

<u>If YES, and READY to start now: Stage 3 (Action)</u>
Summarize the session: So, now you know the recommended levels of use of five dietary components that we have been talking about and also the average levels of consumption of each in your household. You feel that currently you are ready to make changes in this direction now itself. How can we help you start?
How would you describe your confidence to bring about the dietary changes that we discussed in your household?
Support self-efficacy: I congratulate you on being able to arrive at a decision about this for the health of all the members in your household. You can take small steps initially. Don't try to do everything at once. Keep discussing and talking amongst your household members and support each other. If you do run into problems, don't worry. You can contact me in case you require additional help.
Thank you
